# Characterization of *Bacillus subtilis* Viruses vB_BsuM-Goe2 and vB_BsuM-Goe3

**DOI:** 10.3390/v9060146

**Published:** 2017-06-12

**Authors:** Inka M. Willms, Michael Hoppert, Robert Hertel

**Affiliations:** 1 Department of Genomic and Applied Microbiology & Göttingen Genomics Laboratory, Institute of Microbiology and Genetics, University of Goettingen, 37077 Goettingen, Germany; inkamarie.willms@stud.uni-goettingen.de; 2 Department of General Microbiology, Institute of Microbiology and Genetics, University of Goettingen, 37077 Goettingen, Germany; mhopper@gwdg.de

**Keywords:** phage, virus, *Bacillus subtilis*, vB_BsuM-Goe2, vB_BsuM-Goe3, *Spo1virus*, *Bastillevirus*, *Spounavirinae*

## Abstract

The *Spounavirinae* viruses are ubiquitous in nature and have an obligatory virulent lifestyle. They infect *Firmicutes*, a bacterial phylum containing an array of environmental non-pathogenic and pathogenic organisms. To expand the knowledge of this viral subfamily, new strains were isolated and investigated in this study. Here we present two new viruses, vB_BsuM-Goe2 and vB_BsuM-Goe3, isolated from raw sewage and infecting *Bacillus* species. Both were morphologically classified via transmission electron microscopy (TEM) as members of the *Spounavirinae* subfamily belonging to the *Myoviridae* family. Genomic sequencing and analyses allowed further affiliation of vB_BsuM-Goe2 to the SPO1-like virus group and vB_BsuM-Goe3 to the Bastille-like virus group. Experimentally determined adsorption constant, latency period, burst size and host range for both viruses revealed different survival strategies. Thus vB_BsuM-Goe2 seemed to rely on fewer host species compared to vB_BsuM-Goe3, but efficiently recruits those. Stability tests pointed out that both viruses are best preserved in LB-medium or TMK-buffer at 4 or 21 °C, whereas cryopreservation strongly reduced viability.

## 1. Introduction

Prokaryotic viruses infect bacteria (also called bacteriophages or phages [1]) and archaea. Replication occurs via insertion of viral DNA into the host cell in which it is used to form new virus particles. Prokaryotic viruses are of environmental importance as major predators of prokaryotes, thus playing a key role in prokaryotic ecology and evolution [2,3,4]. They are also considered as an alternative treatment method for fighting bacterial infections [5,6].

The *Spounavirinae* subfamily is ubiquitously present in nature. Its members have an obligatory virulent lifestyle and a broad host range. They infect *Firmicutes*, which is a phylum containing an array of environmental non-pathogenic organisms like *Bacillus subtilis* or *Bacillus megaterium*. *Firmicutes* also includes insect pathogens like *Bacillus thuringiensis* as well as *Bacillus cereus* and *Listeria monocytogenes,* which are associated with food poisoning [7]. In addition, clinically relevant bacterial species such as *Bacillus anthracis* [8], *Staphylococcus aureus* and *Clostridium botulinum* [9,10] are members of this phylum. Thus, *Spounavirinae* are promising candidates for the biological control of pathogenic *Firmicutes* [11].

Viruses of this subfamily are large *Myoviridae* with isometric heads of approximately 75–100 nm and long contractile tails ranging from 140 to 220 nm [12]. Recently, the *Spounavirinae* subfamily was further divided into 5 genera (*Kayvirus*, *P100virus*, *Silviavirus*, *Spo1virus* and *Twortvirus*), and a genus *Bastillevirus* outside the subfamily harbouring former *Spounavirinae* members [1]. Silviaviruses, Kayviruses and Twortviruses recruit *Staphylococcus* as a host organism, P100viruses infect *Listeria*, while *Enterococcus* and *Lactobacillus* are recruited by some unassigned viruses. Members of the *Spo1virus* and *Bastillevirus* use *Bacillus* strains as host organisms [1]. Those genera represent the SPO1-like and the Bastille-like groups, which further unite related virus strains [13].

The aim of this study was to expand knowledge on the *Spounavirinae* subfamily via isolation and characterisation of new strains. Therefore, *B. subtilis* ∆6 [14] was used as host strain to isolate vB_BsuM-Goe2 and vB_BsuM-Goe3. Both viruses were morphologically, genomically and functionally investigated. The results allowed a phylogenetic classification and assessment of the survival strategy of both viruses.

## 2. Material and Methods

### 2.1. Naming

Isolates were named based on the systematic scheme suggested by Kropinski, Prangishvili and Lavigne 2009 [15]. Accordingly, vB stands for virus of bacteria, Bsu for the host organism *B. subtilis*, M for the virus family *Myoviridae* and finally Goe2 and Goe3 for the individual name of the isolates. Consequently, the full names of the viruses compose to vB_BsuM-Goe2 and vB_BsuM-Goe3, abbreviated Goe2 and Goe3.

### 2.2. Media and Buffers

LB-medium (10 g peptone, 5 g yeast extract, 10 g NaCl_2_ in 1 L H_2_O) served as nutrient broth for bacterial growth. For agar plates LB medium was solidified with 1.5% (*w*/*v*) agar (Carl Roth GmbH + Co., KG, Karlsruhe, Germany). Overlays for plaque assays consisted of LB medium supplemented with 0.8% (*w*/*v*) low-melting plaque agarose (Biozym Scientific GmbH, Hess. Oldendorf, Germany). TMK buffer [16] consisted of 0.01 M Tris, 0.005 M MgCl_2_ and 0.3 M KCl (pH 7.5), and TN buffer of 20 mM Tris-HCl, and 50 mM NaCl (pH 7.5).

### 2.3. Virus Isolation and Preparation

*Bacillus subtilis* Δ6 [14], a derivate of the model strain *B. subtilis* 168 [17], served as host for virus isolation. Raw sewage from a municipal sewage treatment plant in Göttingen (Germany, 51°33′15.4″ N 9°55′06.4″ E) was used as inoculum. The isolation and propagation procedure were performed as described previously [18]. Plaque assays were performed by mixing 100 µL of the overnight host culture with 100 µL of virus suspension and incubating for 5 min at room temperature. The bacteria-virus suspension was subsequently mixed with 2 mL LB and 2 mL of 0.8% low-melting plaque agarose (40 °C) (Biozym Scientific GmbH, Hess.) dissolved in LB medium and spread on pre-warmed LB plates. After solidification of the agarose, plates were incubated at 30 °C overnight. Plaque morphology was monitored with a stereo microscope in transmitted light mode. Countable range of plaques per 9 cm diameter petri-dish was set from 30 to 1000 for Goe2 and from 30 to 1500 plaques for Goe3, due to the smaller plaque size of Goe3 compared to the plaques of Goe2.

### 2.4. Transmission Electron Microscopy

Negative-staining and transmission electron microscopy were performed as previously described [19]. Phosphotungstic acid dissolved in pure water (3% *w*/*v*) and adjusted to pH 7 served as staining solution. Electron microscopy was carried out on a Jeol 1011 electron microscope (Eching, Munich, Germany). Images were captured using a Gatan Orius 4 K camera and processed with the Gatan 314 Digital Micrograph software package (Gatan, GmbH, Munich, Germany) and Adobe Photoshop CS2 (Adobe Systems Inc., San José, CA, USA). Determination of the average sizes of each isolate base on the dimensions of, at least, six individual virions.

### 2.5. Host Range and Sensitivity Determination

The host range of Goe2 and Goe3 was determined via plaque assays described for virus isolation and spot test assay. For the latter, concentrated virion solution was dropped on three areas of the overlay plates with different strains (see Table 1) and incubated at 30 °C for 16 h. Bacterial strains developing decreased turbidity in the area exposed to the virion solution were considered sensitive to the respective virus. The experiment was performed in triplicate.

### 2.6. Adsorption Constant K

Adsorption constant k determination was based on the instruction by Shao and Wang 2008 [20]. First, living cells mL^−1^ with respect to different optical density at 600 nm (OD_600_) were determined by counting colony forming units (CFU) on LB agar plates. A calibration curve with the following formula was calculated

CFU mL^−1^ = 9 × 10^7^·OD_600_ + 4 × 10^6^

Accordingly, a culture with an OD_600_ of 1 contains approximately 10^8^ cells mL^−1^. Afterwards, 30 reaction vials with 4 mL LB were inoculated up to an OD_600_ of 0.1. They were incubated at 30 °C under vigorous shaking up to an OD_600_ of 1. Subsequently, they were infected with the virus isolates to a multiplicity of infection (MOI) of 0.01. The infection process was frequently interrupted by sterile filtration of the bacteria virus solution. To determine the amount of un-adsorbed virus particles the filtrate was used for overlay plaque assays. A bacteria-free setup served as control.

To calculate the adsorption constant, the logarithm of the plaques counted at the beginning, divided by the plaques counted at time *t* (log$\frac{P_{0}}{P_{t}}$), were plotted against the time *t*. A linear regression curve was drawn. The adsorption constant *k* was calculated by dividing the negative slope of the regression (*m*) with the amount of bacterial cells (*N*), as described by Hyman and Abedon 2009 [21]:

k=−mN

### 2.7. Latency Period

The latency period was determined by a turbidimetric assay with a synergy 2 microplate reader (BioTek, Winooski, VT, USA). 150 µL of fresh bacterial cultures with an OD_600_ of 1 were transferred into the wells of a sterile 96 well-plate. Cultures were either infected with a MOI of 1, 5 or 100 and cell density was directly monitored for 4 h 45 min. The OD_600_ was determined every 2 min with 8 measurements calculated into one average value. Between the measurements cultures were shaken at medium speed. The utilized software for data assessment was Gen5 version 2.09 (BioTek). The average optical density of the triplicates was calculated and plotted over time t in RStudio version 0.99.902 [22] with R version 3.3.1 [23]. A locally weighted least squares regression method was applied with the loess.smooth function from the R stat package in order to average the data distribution.

### 2.8. Burst Size

The burst size of Goe2 and Goe3 was determined with an approach, focusing on the time before and after cell burst, based on the recommendations of Hyman and Abedon 2009 [21]. For the determination of the individual parameters, a fresh *B. subtilis* Δ6 culture with an OD_600_ of 1.0 was infected with a MOI of 0.1, regarding Goe2 and with a MOI of 1, regarding Goe3. After 10 min of adsorption, the infected cultures were diluted to 1:10^3^ for Goe2 or up to 1:10^4^ for Goe3 with pre-warmed LB-medium in order to synchronize the infection [21]. For determination of un-adsorbed virions, an equivalent culture was sterile filtered and free virions quantified via plaque assay. The number of successfully infected host cells was calculated by subtraction of free virions after adsorption from initially introduced virions. The result equals the number of infected cells. The amount of virus progeny was determined via overlay plating of the infected cultures after 140 min and 150 min for Goe2, and 95 min and 105 min for Goe3. A result was accepted as solid when the overlay plates of the different time points showed a consistent number of plaques, validating a plateau. The precise number of virion progeny was calculated via subtraction of the un-adsorbed virions from the determined offspring plaque number. All experiments were performed with 3–6 replicates and all titration steps were performed twice. To calculate the burst size, the following equation of Bolger-Munro [24] was applied:

$$\text{average}\;\text{burst}\;\text{size}=\frac{\left(offspring\;plaque\;number\;-\;initially\;unadsorped\;virions\right)}{\left(introduced\;virions\;-\;initially\;unadsorped\;virions\right)}$$

### 2.9. Simulation of a Virus Infection

Bacterial lysis was simulated in a theoretical cell culture of 10^8^ cells mL^−1^, infected with a MOI of 1. The infection was not considered to be synchronized and the model covers 100 min starting directly after infection. The program used for the calculations was R 3.3.1 [23]. The amounts of newly infected bacteria were calculated for each minute after infection. Thereby, the decreasing amount of free virions throughout the time after infection was considered, as well as the bacterial growth. The latter was introduced into the model by multiplying the growth rate of the bacteria in the exponential phase (0.0115 replications min^−1^) with the number of uninfected cells. To consider cell burst, the amount of newly infected bacteria from the first infection cycle was subtracted from all bacteria in the second infection cycle. The number of newly released virions was calculated via multiplication of the burst size with the amount of new infections per minute during the first infection cycle.

The following two equations described by Storpar and Abedon [25] were used to calculate the simulation:

$$N_{infected\;bacteria}=N_{all\;bacteria}-(N_{all\;bacteria}\cdot \left(1-e^{-k\cdot N_{free\;virions}\cdot 1\min}\right)$$

$$N_{free\;virions}=N_{virions}\cdot e^{-k\cdot N_{all\;bacteria}\cdot 1\min}$$

The bacterial cell density was plotted against the time after infection.

### 2.10. Viability Testing

The viability of Goe2 and Goe3 was tested in LB, TN and TMK buffer (composition described above) at 21 °C, 4 °C and −80 °C. At −80 °C virus suspensions were stored with glycerol (20% *v*/*v*). Freshly prepared stocks were titered and used to make 1 mL virus solutions with a concentration of 10^5^ plaque forming units (PFU) mL^−1^. Virion concentrations were ascertained via overlay plaque assays with 10 µL of the prepared solutions. Experiments were performed in triplicate and data were processed in RStudio version 0.99.902 [22] with R 3.1.1 [23]. The average and standard deviation of the triplicates were calculated. A locally weighted least squares regression method was applied with the loess.smooth function from the R stat package in order to average the data distribution.

### 2.11. Genome Sequencing and Annotation

Virus DNA was isolated as described previously [19] using the MasterPure complete DNA and RNA purification kit (Epicentre, Madison, WI, USA). Libraries for Illumina sequencing were generated with the Nextera XT DNA sample preparation kit, as recommended by the manufacturer (Illumina, San Diego, CA, USA). Sequencing was performed with the Illumina MiSeq sequencer, applying the paired end protocol (2 × 300 base reads) and the MiSeq reagent kit version 3 (Illumina).

**Figure 1:**
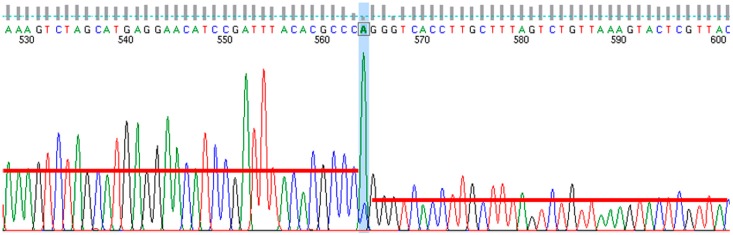
Sequence intensity graph at Long Terminal Repeat (LTR) border. The LTR end is marked through the adenine peak highlighted in blue. It covers a smaller cytosine peak, which is the first base of the non-repeated genome between both LTRs. Downstream of the adenine peak, the sequence intensity graph drops (red bars) as the template amount for the sequence reaction is halved after the LTR border. The figure is visualized via finchtv_1_3_1 (PerkinElmer Inc.).

Long Terminal Repeats (LTRs) were detected via sequence read mapping on the initially assembled genomes using Bowtie2 [26] with the parameter—very-fast. The resulting mapping was visualized with Tablet 1.12.03.26 [27]. The LTR regions exhibited two times increased coverage. The so-identified and isolated LTR sequence was used as template for a further Bowtie2 mapping with the parameters—very-fast—un-conc, resulting in a set of sequence reads free of LTR reads. This sequence set was used for assembly resulting in a single contig flanked by remnants of the LTR regions. The resulting genome sequence was manually joined with the previously identified LTR sequence using Gap4 [28]. The lengths of the LTRs were determined via primer walking using Sanger sequence technology. Borders of the LTRs were detected by the drop of signal intensity at the end of the LTR, as well as by a prominent adenine peak (see Figure 1). The Sanger quality graph was visualized with finchtv_1_3_1 (PerkinElmer Inc., Waltham, MA, USA). The adenine peak is a Taq-polymerase related artefact, as this polymerase adds a template-free adenine to the end of a sequence. The sequence between the two adenine peaks was used to define the exact genome size.

Genome maps (see below in Figure 4) were visualized via DNA plotter with the software Artemis 16.00 [29] and further processed with Adobe Illustrator CS2 (Adobe Systems Inc.). Global alignments of phage genomes were visualized with Easyfig 2.2.2. [29] and further processed with Adobe Illustrator CS2 (Adobe Systems Inc.). 

Genomes were automatically annotated using PROKKA v.1.11 [30], PHAST [31] and tRNAscan-SE v.1.2 [32]. All results were combined manually. At sites of discrepancies a blastp [33] analysis via the NCBI web interface, using the non-redundant database, and an InterProScan [34] sequence search via the European Bioinformatics Institute (EBI) web interface was performed.

The annotated genome sequences were submitted to GeneBank and are available under the accession no. KY368639 (Goe2) and KY368640 (Goe3). Corresponding strains were deposited at the German Collection of Microorganisms and Cell Cultures (DSMZ) where they are available as strain DSM 105105 (Goe2) and DSM 105106 (Goe3).

### 2.12. Phylogeny

Similarities of Goe2 and Goe3 genomes to other genomes were determined through global blastn [33] using the non-redundant nucleotide NCBI database. The genomes of the closest relatives of both viruses were used, together with those of the type strains of the respective genus, for a global blastx alignment using Easyfig 2.2.2 [35].

For further phylogenetic investigations of Goe2 and Goe3, a Maximum Likelihood tree was calculated using the major capsid protein (Goe2_c07600, Goe3_c07100) and the thymidylate synthase (Goe2_c13700, Goe3_c03500) as marker genes. Strains were used for comparison according to Asare et al. [13]. The evolutionary history was inferred by using the Maximum Likelihood method based on the JTT matrix-based model [36]. The trees with the highest log likelihood are shown below in Figure 6. The percentage of trees in which the associated taxa clustered together is shown next to the branches. Initial tree(s) for the heuristic search were obtained automatically by applying Neighbor-Join and BioNJ algorithms to a matrix of pairwise distances estimated using a JTT model, and then selecting the topology with superior log likelihood value. The trees are drawn to scale, with branch lengths measured in the number of substitutions per site. The analysis involved 34 amino acid sequences each. All positions containing gaps and missing data were eliminated. Evolutionary analyses were conducted in MEGA7 [37] and untrimmed sequences were aligned with the integrated program Muscle [38]. The trees were calculated with 500 iterations.

## 3. Results

### 3.1. Virus Isolation

**Figure 2:**
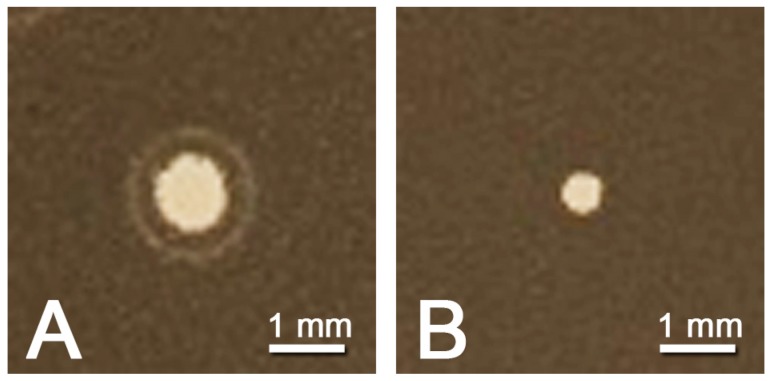
Plaque morphology of vB_BsuM-Goe2 (Goe2) and vB_BsuM-Goe3 (Goe3) on *Bacillus subtilis* Δ6 overlay plates. (A) Goe2 exhibits a plaque diameter of 1.1 mm with a halo of decreased turbidity on its periphery; (B) Goe3 exhibits a plaque diameter of about 0.6 mm in diameter.

The viruses Goe2 and Goe3 were isolated using overlay plaque assays with *B. subtilis* Δ6 [14] as host bacterium. This bacterial strain is a provirus- (or prophage) free derivate of the laboratory strain *B. subtilis* 168 [17]. Both viruses were isolated from the same sample of raw sewage. Virus plaque analysis revealed wider plaques for Goe2 (~1.1 mm) than for Goe3 (~0.6 mm) (see Figure 2). Aside from the increased size, the plaques of Goe2 frequently exhibited a halo on their periphery.

### 3.2. Phage Particle Morphology

**Figure 3:**
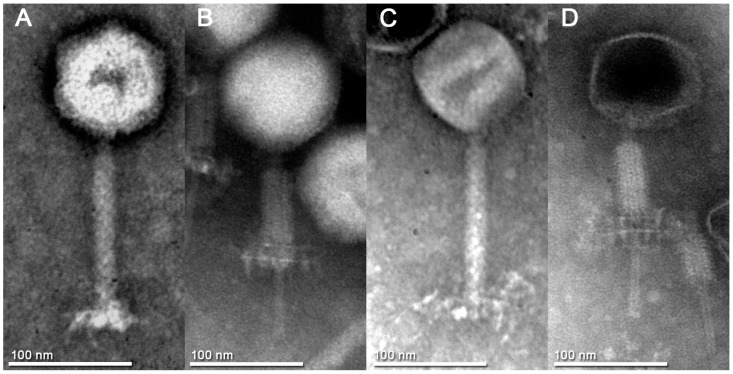
Electron micrographs of Goe2 and Goe3. (**A**) Goe2 in the non-contracted state. The average head capsid is 95.36 ± 2.03 nm wide and 103.09 ± 3.02 nm high, and the tail is 163.37 ± 7.86 nm long and 17.70 ± 1.25 nm wide. (**B**) Goe2 in the contracted state with a visible double base plate. (**C**) Goe3 in the non-contracted state. The average head capsid is 95.74 ± 3.90 nm wide and 98.36 ± 4.53 nm high, and the tail is 163.05 ± 3.41 nm long and 17.87 ± 0.87 nm wide. (**D**) Goe3 in the contracted state with a visible double baseplate. An isometric head capsid and tail fibres, attached to the baseplate beneath the tail are exhibited by both viruses. Goe3 (**C**) has longer tail-fibres than Goe2 (**A**). 3% phosphotungstic acid was used for negative staining of the virions.

The morphology of Goe2 and Goe3 was analysed via transmission electron microscopy (TEM). The TEM micrographs are shown in Figure 3. Both virus particles exhibited a head and tail morphology, which is typical for members of the order *Caudovirales.* Furthermore, a long contractile tail was detected, characteristic of the *Myoviridae* family. Additional morphological features shared by both viruses were the isometric head capsid and the tail fibres attached beneath the baseplate, at the end of the tail protein. Both virions experienced a morphological change upon tail sheath contraction in the form of a double base plate (Figure 3B,D). All morphological properties support the assignment of Goe2 and Goe3 to the *Spounavirinae* subfamily [12]. The head of an intact Goe2 virion is about 95 nm wide and 103 nm high, and the tail 163 nm long and 18 nm wide. The head of an intact Goe3 virion is about 96 nm wide and 98 nm high, and the tail 163 nm long and 18 nm wide. Tail fibres of Goe3 are considerably longer than those of Goe2 (see Figure 3A,C).

### 3.3. Genomic Structure and Content

**Figure 4:**
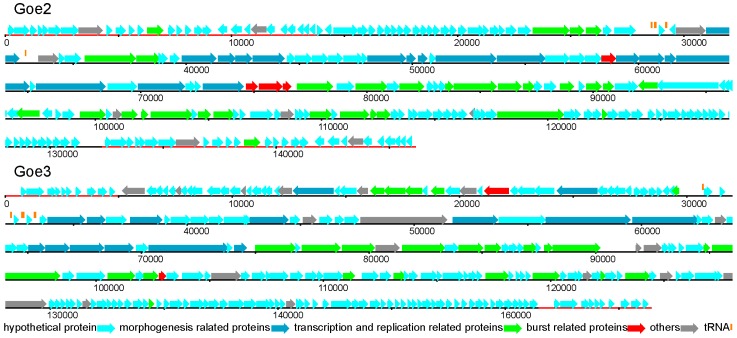
Genomic overview of Goe2 and Goe3. Genes are coloured in relation to their annotation. LTRs are marked as red lines. Goe2 has longer LTRs (13.85 kbps) compared to Goe3 (4.954 kbps), whereas Goe3 discloses a larger genome (156.430 kbps) with 5 tRNAs and 246 protein coding regions compared to Goe2 (146.141 kbps) with 4 tRNAs and 226 protein coding regions.

The genomes of Goe2 and Goe3 are linear and have LTRs flanking the genome. These findings are in good agreement with the morphology-based classification of both viruses as members of the *Spounavirinae* subfamily [12]. Based on the sequence results the genome of Goe2 consists of 146.141 kbp including LTRs of 13.85 kbp on both ends, and a GC content of 40.26%. Genome analysis revealed the presence of 4 tRNAs and 226 predicted protein-encoding genes, of which 165 code for hypothetical proteins, 4 for burst related, 31 for transcription and replication related and 17 for morphogenesis related proteins. Eleven regions code for proteins with other predicted functions (see Figure 4).

The genome of Goe3 consists of 156.430 kbp with LTRs of 4.954 kbp on both genome ends, and a GC content of 41.93%. It contains 5 tRNAs and 246 predicted protein-encoding genes of which 186 code for hypothetical proteins, 2 for burst related, 25 for transcription and replication related and 15 for morphogenesis related proteins. Eighteen genes code for proteins with other functions (see Figure 4). From the last mentioned group one protein gene codes for a poly-gamma-glutamate hydrolase (Goe3_c02900). This enzyme is not directly associated with virus replication, but is beneficial for further host infection as it disintegrates the biofilm matrix and therefore ensures a better access to suitable hosts [39]. A similar gene was found in the Bastille-like virus phiNIT1 (accession number: PRJNA213017) indicating a frequent occurrence.

### 3.4. Phylogeny and Classification

**Figure 5:**
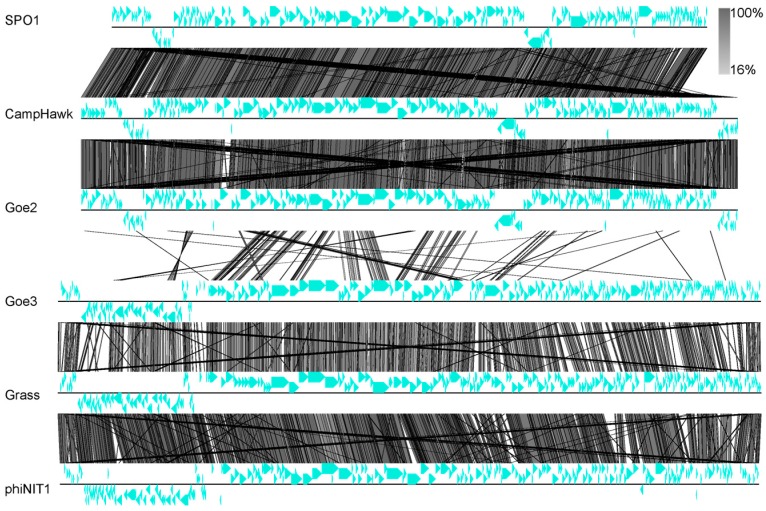
Pairwise genome alignment of SPO1, CampHawk, Goe2, Goe3, Grass and phiNIT1. Blue arrows indicate protein coding genes and their orientation. Vertical grey lines indicate blastx similarities between compared genomes. Similarity to multiple regions leads to multiple lines from the seed region. The degree of similarity is represented by the grey level. Similarity key is presented in the figure. Goe2 and Goe3 resemble only slightly on the tblastx comparison, whereas there is a higher similarity to their nearest relatives. Goe3 shares less similarity to Grass and phiNIT1 than Goe2 to CampHawk and SPO1.

Regarding the recommendation of the International Committee on Taxonomy of Viruses (ICTV) [1], further classification focused on blast-based investigation. Similarity searches of Goe2 on nucleotide level revealed sequence identities of 96% to the *Bacillus* viruses CampHawk [40] and SPO1 [41] with a coverage of 97% and 95% respectively. The genome of Goe3 showed the highest identity to *Bacillus* viruses Grass [42] and phiNIT1 (accession number: PRJNA213017) with sequence identities of 79% and 77%, respectively, but with a genome coverage of only 23% and 21%, respectively. Asare et al. [13] recently demonstrated that CampHawk and SPO1 belong to the SPO1-like virus group, whereas phiNIT1 belongs to the Bastille-like virus group. The authors defined the presence of a dihydrofolate reductase and/or a protein similar to a DNA translocase of stage III sporulation of *B. cereus*, as specific markers for the Bastille-like virus group. Both marker genes could be identified in the genome of Goe3 (Goe3_c03300, Goe3_c17800). Considering all described results, we propose Goe2 as a member of the SPO1-like virus group and Goe3 as a member of the Bastille-like virus group. This is further supported by overall genomic similarities of Goe2 and Goe3 to their closest related viruses and their differences between each other determined via blastx (see Figure 5). The complementary regions of Goe2, CampHawk and SPO1 are in analogous locations and show similar gene arrangements and orientations. In contrast to Goe2, the Goe3 genome reveals smaller regions with high similarity to its closest relatives and is therefore less alike to Grass and phiNIT1. However, comparable gene arrangements and orientation can also be observed (see Figure 5).

**Figure 6:**
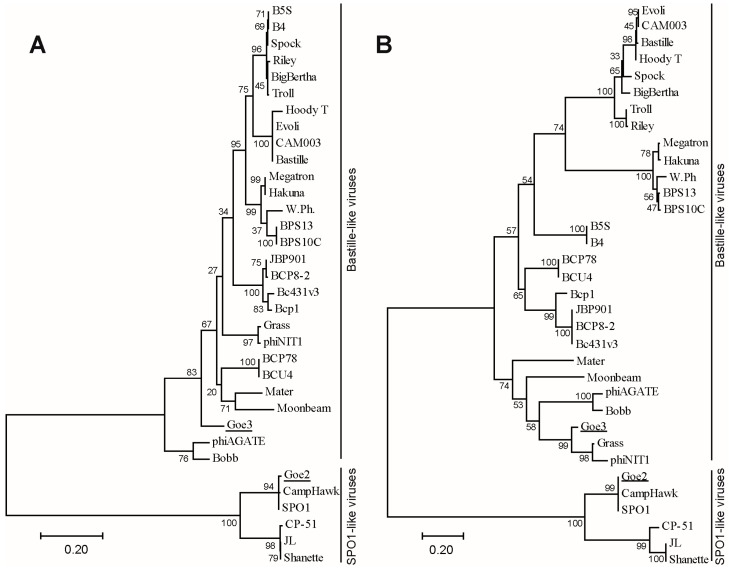
Phylogenetic classification of Goe2 and Goe3. The phylogenetic trees were calculated using (**A**) the major capsid protein of 34 *Bacillus* viruses and (**B**) the thymidylate synthase gene of 34 *Bacillus* viruses, and the Maximum Likelihood method. The trees are drawn to scale, with branch lengths measured in the number of substitutions per site. The percentage of trees in which the associated taxa clustered together is shown next to the branches. Goe2 and Goe3 are underlined. Goe2 clusters into the branch of SPO1-like viruses in close proximity to SPO1 and CampHawk, in both calculations. Goe3 clusters into the Bastille-like virus branch with noticeable distance to Grass and phiNIT1 in (**A**) and in close proximity in (**B**).

To bring Goe2 and Goe3 into a more global context, two phylogenetic trees were calculated with further members of the SPO1-like and the Bastille-like viruses. The genes for the major capsid protein (Goe2_c07600, Goe3_c07100) and the thymidylate synthase (Goe2_c13700, Goe3_c03500) served as markers for this investigation. Figure 6 reveals Goe2 clustering with its closest relatives, identified by blastn in both phylogenetic trees. Goe3 clusters with the most similar viruses Grass and phiNIT1 into the Bastille-like virus group, based on the thymidylate synthase. However it demonstrates greater distance to both in the major capsid protein calculations.

### 3.5. Virus Host Interaction

**Figure 7:**
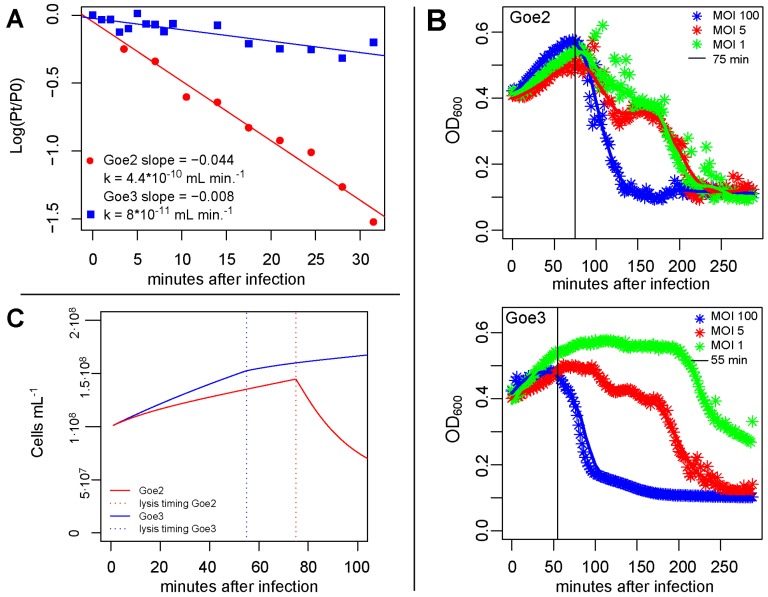
Physiological parameter of Goe2 and Goe3. (**A**) Adsorption constant of Goe2 and Goe3 on *Bacillus subtilis* Δ6. The adsorption constant was determined via the infection of 10^8^ cells mL^−1^ with Goe2 or Goe3 (multiplicity of infection (MOI) 0.01), in LB-medium at 30 °C. The logarithm of the free phage amount at time point 0 divided by free phage counts at later time points is plotted on the linear *y*-axis against the time in minutes after infection. The slope of the regression line is −0.044 for Goe2 and −0.008 for Goe3. Based on the slopes, the adsorption constant of Goe2 was calculated to be 4.4 × 10^−10^ whereas Goe3 has an adsorption constant of 8 × 10^−11^ mL·min^−1^. (**B**) Latency period of Goe2 and Goe3 on *B. subtilis* Δ6. Each asterisk represents the average of 8 measurements of three replicates performed every second minute. The solid lines are regression lines generated via the scatter.loess function in R. The blue dataset represents the cultures infected with Goe2 or Goe3 at a MOI 100, the red represents those infected with a MOI 5 and the green those infected with MOI 1. The horizontal line displays the determined latency time. For Goe2 the latency time is 75 min and in the case of Goe3 55 min. (**C**) Simulation of virus/host interaction. The solid lines represent the cells of cultures infected with Goe2 (red) or Goe3 (blue), at MOI 1. The dashed horizontal lines represent the time of lysis. Goe2- and Goe3-infected cultures experienced an increase of cell density during the first infection cycle, whereas Goe3-infected cultures grew more effectively. The increase in cell density of Goe3-infected cultures was reduced during the second infection cycle, but was still positive. Goe2-infected cultures significantly decreased in cell density during the second infection cycle.

The adsorption constant k of Goe2 and Goe3 was determined by monitoring the decrease in free virions in a *B. subtilis* Δ6 culture over time (31.5 min). Figure 7A depicts the logarithm of the amount of free virions at time 0 divided by the amount of free virions at later time points. Linear regression analysis revealed for Goe2 and Goe3 slopes of −0.044 and −0.008, and adsorption constants of 4.4 × 10^−10^ and of 8 × 10^−11^ mL·min.^−1^, respectively. Thus, the adsorption efficacy to a host cell of Goe2 is 5.5-fold higher than of Goe3.

Latency periods were determined with a turbidimetric approach, monitoring the decrease in cell density due to cell lysis from virus infection. Bacterial cultures were infected with a multiplicity of infection (MOI) of 1, 5 and 100. The cultures infected with MOI 100 point out the latency period best (see Figure 7B). Cultures infected with MOI 5 and MOI 1 further confirmed the latency period via a decrease in cell density during the second and third infection cycles. Consequently, the latency period of Goe2 is 75 min and 55 min for Goe3.

After latency periods were determined, it was possible to calculate the burst sizes of both viruses by monitoring a synchronized infection. This was carried out through a stand-alone approach with emphasis on the time before and after cell burst. Based on this experimental procedure, the average burst size of Goe2 was calculated to be 142 virions/burst and 114 virions/burst for Goe3.

All physiological parameters of Goe2 and Goe3 were combined in a simulation for cross verification as all were determined independently from each other. Thus, a bacterial infection of 10^8^ cells mL^−1^ with a MOI 1 was simulated in R [23]. During the first infection cycle, an increase of cell density was calculated in both theoretical cultures. After the first burst of Goe3 the infected culture still exhibited growth, whereas the cell density of the Goe2-infected culture noticeably decreased (see Figure 7C). This simulation is in good agreement with the actual trend of the monitored cell density during the determination of the latency period (see Figure 7B), and thus cross verifies the experimentally determined physiological parameters.

### 3.6. Host Range and Sensitivity Determination

The host range of both viruses was determined via a plaque assay and sensitive strains were identified with a spot test assay. For this purpose, *B. subtilis* 168 [43], *B. subtilis* NCIB3610 [44], *B. subtilis* natto [45], *Bacillus licheniformis* DSM13 [46,47], *B. licheniformis* 9945A [48], *B. licheniformis* 12369 [49], *B. amyloliquefaciens* FZB42 [50] and *Bacillus pumilus* SAFR-032 [51] served as test strains. 

Defined plaques could be observed only on *Bacillus amyloliquefaciens* FZB42, where both viruses demonstrated plaque size and morphology similar to plaques on *B. subtilis* Δ6. All other strains revealed no clear plaques under the applied experimental conditions. This was not surprising as Goe3 itself was initially identified not as a plaque, but as an almost invisible tiny glossy spot on a matt bacterial layer. Only after some optimisation efforts, was it possible to generate defined plaques with this virus. To overcome the optimisation procedure for each potential host strain a sensitivity test was performed via a spot test assay. Even though this type of experiment does not certainly clarify the potential host range, it still gives an overview of strains sensitive to the exposed viruses. All tested strains showed sensitivity to Goe3, whereas Goe2 was not able to lyse the cells of *B. licheniformis* DSM13, *B. licheniformis* 12369 and *B. pumilus* SAFR-032. However, when lytic activity was detected for both viruses, Goe2 caused stronger reduction of turbidity, except for *B. amyloliquefaciens* FZB42, where both viruses showed the same effect (see Table 1).

### 3.7. Viability Testing

**Figure 8:**
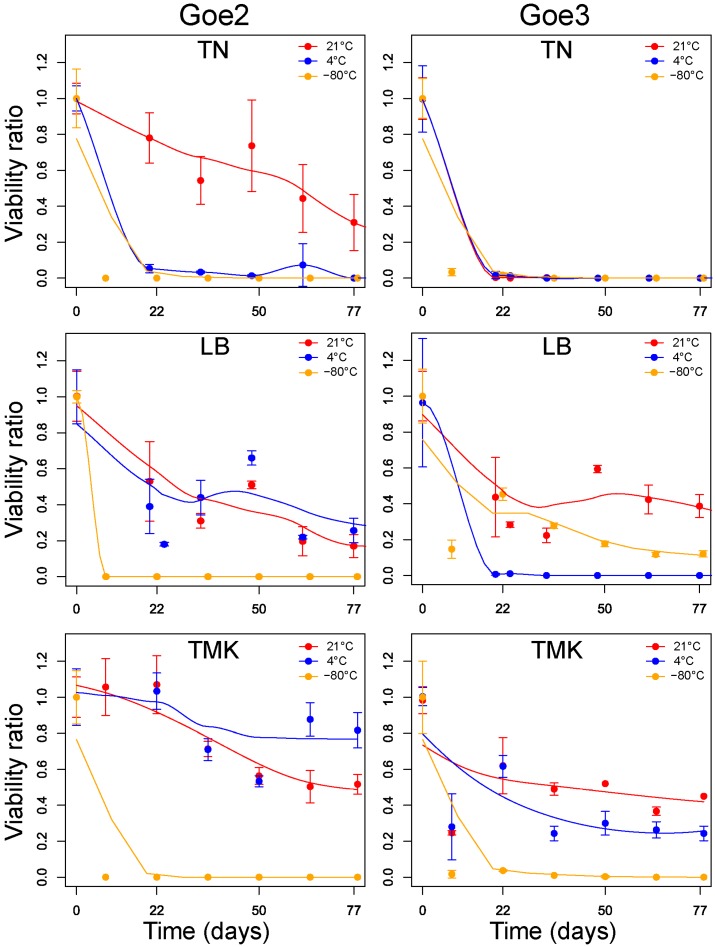
Viability test of Goe2 and Goe3. Virion viability was determined via plaque overlay assay. Each data point is the average of three replicates. The standard deviation is shown through error bars. Data determined at 21 °C are presented in red, at 4 °C in blue and at −80 °C in orange. For data regression the loess.smooth function in R was used.

Particle viability was examined in LB-medium, TMK buffer and TN buffer, at 21 °C, 4 °C and −80 °C over 2.5 months. Both viruses were very unstable when stored in TN buffer regardless of the temperature (see Figure 8), as they lost up to 100% of the initial infection potential after the first 3 weeks of storage. Exclusively, Goe2 stored at 21 °C in TN buffer did not experience total inactivation during the investigation. Higher stability was observed in LB medium for both viruses but a decrease of virus titers was still apparent in all conditions. However, storage in LB medium at 21 °C stabilized at about 20% for Goe2 and at about 30% for Goe3 after 9 weeks. The highest viability was observed in TMK buffer (see Figure 8). Goe2 stabilized at about 80% viability at 4 °C and 45% at 21 °C, while Goe3 stabilized at about 40% at 21 °C and 30% at 4 °C. This might be attributed to the divalent magnesium ions and additional potassium ions present in the buffer. In general, storage of both viruses as a cryogenic culture at −80 °C was not efficient, regardless of the storage solution.

## 4. Discussion

The *Bacillus* viruses Goe2 and Goe3 were simultaneously isolated from a sewage sample. Physiology of both viruses appeared to be distinct as different plaque sizes were observed, initially (see Figure 2). The development and size of a plaque depends mainly on virion diffusion from the initial infection. How fast that happens, in turn, depends on phage intrinsic properties such as particle size and agar overlay density [52]. Variety on virion morphology was expected as both faced the same experimental conditions. Therefore it was surprising to recover almost identical virion particles via TEM (see Figure 3), which stated that size was not responsible for the observed plaque morphologies. However, further virus classification, based on genome comparison with closely related viruses, indicated each isolate to be associated with a distinct viral group. Goe2 is most likely a member of the SPO1-like viruses group closely related to CampHawk [40] and SPO1 [41], and Goe3 is most likely a member of the Bastille-like virus group, such as Grass and phiNIT1 (see Figure 5). To our knowledge, members of these groups have never been directly compared on a physiological level, in which, at least, the viruses presented here obviously differ. For this reason, this investigation of Goe2 and Goe3 presents a first survey of comparable physiological data of viruses associated with the SPO1-like and Bastille-like viruses.

Beside their morphology both isolates clearly differed in all determined parameters. This was most obvious in the case of adsorption time. Goe2 proved to associate with its host cell 5.5 times more efficiently as Goe3. On the other hand Goe3 proved to be superior concerning the latency period; however, this advantage most likely is balanced out by the reduced offspring number compared to Goe2. Thus, Goe3 could not compete with Goe2 under the applied experimental conditions. This became evident during the latency period determination (see Figure 7B) where Goe3 needed a MOI 100 for a successful result, whereas Goe2 succeeded with MOI 1. The reduced adsorption efficiency of Goe3 compared to Goe2 could also explain the difference in the initially observed plaques. Virion size is not the only plaque size determining factor [52]. Particles with reduced adsorption ability can diffuse from their release point without attaching to the next available host and, therefore, do not contribute to plaque enlargement [52] which leads to the smaller plaques of Goe3. Goe2, in contrast, has advanced adsorption ability to its host, which leads to larger plaques and probably also explains the frequently observed inhomogeneous plaques. The so-called “bull’s eye plaque” may be a result of lysis inhibition caused by further adsorption of additional viruses [53]. Such a phenomenon was described for T-even viruses, where it led to a longer latency period and larger burst size [53]. In such a case, the turbid ring around the clear Goe2 plaque would represent a region with multiple infections, as a consequence of superior adsorption ability, which resulted in a prolonged latency period. The following clear ring would explain itself as a zone with sufficiently reduced virion concentration to cause normal progression.

Despite the clear advantage of Goe2 over Goe3 in the applied experimental conditions, it is reasonable to assume that in a natural environment, Goe3 can successfully maintain itself. Natural habitats are generally of higher heterogeneity compared to an experimental setup with a single strain. Nutrients are limited and different bacteria compete for the same resource. The experimentally verified broader spectrum of sensitive bacterial strains for Goe3 indicates a potentially broader host range (see Table 1) and may be the key to its success. According to Ashelford et al. [54] longer latency periods and larger burst sizes correlate with sparse host bacteria, which could hold true for Goe2, as it has a potentially narrower host range compared to Goe3. On the other hand, short latency periods and small burst sizes are typical for viruses populating an environment with high host cell quantity, which could be true for Goe3 as it showed a broader range of sensitive strains compared to Goe2. Additionally, Wang et al. [55] stated that a high host cell density is equivalent to a virus population with a high adsorption constant, as both factors reduce the average time to find a host. Taking everything into account, the observed inferiority of Goe3 to Goe2 under artificial experimental conditions most likely originates from a different survival strategy.

### 4.1. Comparability of Physiological Data

The presented physiological data of Goe2 and Goe3 indicate distinct ecological strategies, which may also apply to the groups each virus is associated with. To further prove this assumption, more comparable data is required. Unfortunately, existing data is rare and often incomplete, as demonstrated in Table 2, where the few available physiological parameters from the 34 strains, used for phylogenetic investigation (see Figure 6), are presented. Furthermore, these properties are often determined under distinct experimental conditions, which make a comparison difficult. For example, it was demonstrated for BCP8-2 [56], that media composition has a considerable influence on the host virus interaction. Investigation on SPO1 also showed that cultivation temperature strongly influences latency period and burst size [57]. During this investigation it became obvious, that methods, used to determine the phenotypical parameter, also have a considerable impact on the final result. For instance, the latency period and burst size of Goe2, determined with the common “one-step growth curve approach” [58], were about 100 min and 6 virus progeny per burst. This differed greatly to the final results which were cross verified via a simulation (see Figure 7C). All in all, this demonstrates the importance and need of comparable datasets to further explore viral ecology. In this regard, the here presented results are a good start point. Further, they nicely demonstrate that bacterial viruses are not just simple machines waiting to be triggered to process their reproduction program. A simple look at their physiology, as was done here, has already revealed differences and potential specialisations for certain niches. Moreover, it was shown recently that these metabolism-free entities are also able to communicate with each other, and make a common lifestyle decision for their population [59]. Therefore, it is unavoidable for the understanding of a virus’s role in the complex environment, to not only investigate a sequenced genome, but also the phonotype itself; the “living” reflection of its genes.

### 4.2. Know Your Phage

Taking the advice “Know your phage” by H.W. Ackermann and colleagues [64] into account a viability test was performed. Results were strongly convincing, independent of the limited experimental period, and clearly stated that Goe2 and Goe3 should not be cryogenically conserved. Storage in TMK buffer turned out as the best solution for long term conservation. However, this requires re-buffering and is unavoidably connected to the loss of biological material. Therefore, sample storage as sterile filtered LB-medium lysates at 4 or 21 °C is a good option. The effect of lyophilisation on sample viability was not investigated due to technical limitations; however, this technique could be an option for a public strain collection.

## 5. Conclusions

With Goe2 and Goe3 two new viruses of *B. subtilis* were isolated. The value of a genome-based classification, in addition to a morphological approach, was demonstrated during the classification of Goe2 as a member of the SPO1-like virus group, and Goe3 as a member of Bastille-like virus group. Experimentally determined physiological parameters revealed their worth by uncovering different survival strategies for both viruses. To our knowledge, the setup applied here allowed a functional comparison between both virus groups for the first time.

## Figures and Tables

**Table 1:** Sensitivity of *Bacillus* species towards vB_BsuM-Goe2 (Goe2) and vB_BsuM-Goe3 (Goe3).

Bacterial Species	Goe2	Goe3
*B. amyloliquefaciens* FZB42	++ P	++ P
*B. licheniformis* 9945A	+	+ −
*B. licheniformis* DSM13	−	+ −
*B. licheniformis* 12369	−	+
*B. pumilus* SAFR-032	−	+ −
*B. subtilis natto*	++	+
*B. subtilis* 168	++	+
*B. subtilis* 3610	++	+
*B. subtilis* Δ6	++ P	+ P

Strains sensitive to the applied viruses revealed reduced bacterial cell density in the applied area of the spot test assay. The following graduations were observed: ++ complete clear spots in the bacterial lawn; + noticeable decrease in turbidity but not complete clear zones; + − minor reduction of turbidity; − no effect on turbidity of the bacterial lawn. Strains marked with “P” revealed plaques on a plaque assay.

**Table 2:** Physiological properties of Bastille- and SPO1-like viruses.

Strain	Host	Burst Size in PFU	Latency Period in min	Adsorption in mL·min^−1^	Reference
B4	*B. cereus*	>200	10–15 min	–	[60]
Bc431v3	*B. cereus*	318	85 min	–	[61]
BCP8-2	*B. cereus*	50	–	–	[56]
BCP1	*B. cereus*	50	–	–	[56]
phiAGATA	*B. pumilus*	153	35	6.44 × 10^−9^	[62]
SPO1	*B. subtilis*	70	80	–	[57]
CP-51	*B. cereus*	90	90–100	–	[63]

Available physiological properties of Bastille- and SPO1-like viruses presented in Figure 6. PFU = plaque forming units.
